# Predicting Unplanned Readmission Risk in Patients With Cirrhosis: Complication-Aware Dynamic Classifier Selection Approach

**DOI:** 10.2196/63581

**Published:** 2025-09-10

**Authors:** Zixin Shi, Linjun Huang, Xiaomei Xu, Kexue Pu, Qingpeng Zhang, Haolin Wang

**Affiliations:** 1 College of Medical Informatics, Chongqing Medical University, 1 Yixueyuan Road, Yuzhong District, Chongqing, 400016, China, 86 13500303273; 2 Department of Infectious Diseases, Chengdu Fifth People's Hospital, Chengdu, China; 3 Musketeers Foundation Institute of Data Science, University of Hong Kong, Hong Kong, China (Hong Kong); 4 Department of Pharmacology and Pharmacy, University of Hong Kong, Hong Kong, China (Hong Kong)

**Keywords:** data mining, electronic health records, multiple classifier systems, predictive models, readmission risk, EHR, cirrhosis, gastrointestinal disease, decision-making, framework

## Abstract

**Background:**

Cirrhosis is a leading cause of noncancer deaths in gastrointestinal diseases, resulting in high hospitalization and readmission rates. Early identification of high-risk patients is vital for proactive interventions and improving health care outcomes. However, the quality and integrity of real-world electronic health records (EHRs) limit their utility in developing risk assessment tools.

**Objective:**

Despite the widespread application of classical and ensemble machine learning for EHR-based predictive tasks, the diversity of health conditions among patients and the inherent limitations of the data, such as incompleteness, sparsity, and temporal dynamics, have not been fully addressed. To tackle those challenges, we explored a framework that characterizes patient subgroups and adaptively selects optimal predictive models for each patient on the fly to enable individualized decision support.

**Methods:**

The proposed framework uniquely addresses patient heterogeneity by aligning diverse subgroups with dynamically selected classifiers. First, patient subgroups are generated and characterized using rules indicating medical diagnosis patterns. Next, a meta-learning framework trains a meta-classifier for optimal dynamic model selection, which identifies suitable models for individual patients. Notably, we incorporated a tailored region of competence to refine model selection, specifically accounting for cirrhosis complications. This approach not only enhances predictive performance but also elucidates why individualized predictions are better supported by selected classifiers trained on specific data subsets.

**Results:**

The proposed framework was evaluated for predicting 14-day and 30-day readmission in patients with cirrhosis using multicenter data obtained from 6 hospitals. The final dataset comprised 3307 patients with at least 2 admission records, along with a range of factors including demographic information, complications, and laboratory test results. The proposed framework achieved an average AUC (area under the curve) improvement of 5% and 4% compared to the best baseline models, respectively.

**Conclusions:**

By leveraging the expertise of the most competent classifiers for each patient subgroup, our approach enables interpretable training and dynamic selection of heterogeneous predictive models. This advancement not only improves prediction accuracy but also highlights its considerable potential for clinical applications, facilitating the alignment of diverse patient subgroups with tailored decision-support algorithms.

## Introduction

Cirrhosis, a chronic condition caused by hepatocyte injury and liver fibrosis [1], is often accompanied by complications [2] such as ascites and hepatic encephalopathy, posing severe risks to patients’ health and survival. Patients with cirrhosis typically have high hospitalization rates, prolonged hospital stays, and frequent readmissions [3]. Notably, patients with decompensated cirrhosis are particularly vulnerable, with a 5-year survival rate of merely 14%‐35% [4] and a readmission rate ranging from 10% to 50% within 7-90 days postdischarge [5]. These challenges not only disrupt the continuity of care and degrade patients’ quality of life but also impose a substantial economic and societal burden on hospitals and health care systems [6]. Statistics indicate that the annual economic expenditure attributable to unplanned readmissions from cirrhosis amounts to approximately US $17.4 billion [7]. Given the significant impact of the readmission issue on health care resource allocation [8], the Patient Protection and Affordable Care Act (2010) established reducing hospital readmission rates as a pivotal objective in the reforming of fee-for-service hospital reimbursement policies [9]. Even though the readmission rate is not commonly used as an evaluation metric by hospitals, the global significance of the readmission problem cannot be ignored [10,11].

Research has demonstrated that a substantial proportion, up to 27.1% [12], of readmissions are potentially preventable, highlighting a substantial opportunity for the development of predictive models aimed at identifying high-risk patients. In recent years, electronic health records (EHRs) have gained widespread adoption in both clinical practice and research endeavors, providing a vast and diverse dataset for machine learning algorithms [13]. By harnessing clinical indicators embedded within EHRs, such as laboratory test results and vital signs, researchers have developed numerous predictive models to precisely evaluate patients’ risk of readmission. For instance, Berman et al [14] used comprehensive EHR data sourced from 2 prominent academic medical centers to identify variables predictive of 30-day readmission among patients with liver disease. Similarly, Hu and colleagues [15] conducted an analysis of 30-day and 90-day readmission rates for patients with end-stage liver disease, leveraging EHR data in conjunction with models such as logistic regression, support vector machines, and random forests. These models hold significant potential to enhance our ability to anticipate and mitigate the risk of readmission in vulnerable patient populations [15].

However, the diverse patient population and the inherent challenges of EHR data [16], including high dimensionality, incompleteness, sparsity, and temporal dynamics, pose substantial obstacles for model development. While individual machine learning models have demonstrated effectiveness on specific datasets, they frequently face difficulties in comprehensively addressing these complexities [17]. Multiple classifier systems [18] with dynamic ensemble selection (DES) [19] emerge as a promising approach to address those challenges. DES typically surpasses single models in predictive performance by selecting and integrating diverse classifiers tailored to unknown test instances during the inference process, thereby enhancing predictive accuracy.

The META-DES (dynamic ensemble selection using meta-learning) framework [20] transforms the task of classifier selection into a meta-problem. This approach entails extracting meta-features pertinent to the base classifiers and training a meta-classifier to assess their classification prowess. Nonetheless, when applied to tabular data classification, particularly in predicting readmissions for patients with cirrhosis, the existing DES algorithms do not always outperform state-of-the-art ensemble models. This performance gap seems to stem from 2 challenges. First, the development of predictive models needs to be deeply integrated with the characteristics of specific clinical problems, such as the complexity and diversity of inpatient care. Second, the quality issues of EHR data, such as incompleteness, sparsity, and temporal dynamics, further exacerbate the difficulties in model development.

To address these challenges, this study aims to develop an interpretable framework that dynamically aligns patient subgroups with optimized classifier selection. Specifically, the aim of this study is threefold: (1) to generate and characterize patient subgroups through clinically meaningful rules based on complication and comorbidity patterns; (2) to design a tailored region of competence incorporating medical diagnoses, enabling optimized dynamic selection of patient-specific predictive models; and (3) to establish a paradigm shift from conventional one-size-fits-all approaches to an adaptive methodology that accounts for heterogeneous patient profiles. By integrating medical knowledge with DES, this approach not only improves predictive performance but also provides interpretable insights into the classifier selection rationale for diverse clinical subgroups.

## Methods

### Ethical Considerations

The study received ethics review and approval from the institutional review board of Chongqing Medical University (approval no. 2023098). Due to its retrospective nature, this study required no informed consent. All collected data were fully anonymized with no personally identifiable information included. No financial or material incentives were provided to participants.

### Data Collection

This retrospective prognostic study focused on decompensated liver cirrhosis data sourced from 6 tertiary hospitals in Chongqing, China. The initial cohort encompassed 13,476 patient records from January 2011 to June 2020, identified using cirrhosis diagnostic codes according to the ICD-9 (*International Classification of Diseases*, 9th Revision) or ICD-10 (*International Classification of Diseases*, 10th Revision) systems. We followed the TRIPOD (Transparent Reporting of a Multivariable Prediction Model for Individual Prognosis or Diagnosis) [21] guidelines for reporting cohort studies. To refine our cohort, we applied the following exclusion criteria: (1) patients hospitalized for cancer, malignant tumors, tuberculosis, or HIV (n=1906); (2) patients lost to follow-up or who died during their initial hospitalization (n=151); (3) patients with no recorded readmissions (n=7150); and (4) patients with missing data exceeding 10% (n=1113).

### Variable Description

The refined cohort consisted of 3307 patients, each with at least 1 readmission record. The dataset covered a wide range of variables, including 3 demographic factors, 5 etiological variables, 35 comorbidities and complications, 38 clinical test indicators, 10 surgical variables, 7 medication-related factors, 1 composite scoring variable, and 1 outcome variable. All included indicators were thoroughly reviewed by clinical experts. Demographic data included age, gender, and the number of hospitalizations. The list of complications featured hepatic encephalopathy, acute-on-chronic liver failure, ascites, and esophageal or gastric varices. The laboratory test indicators included prothrombin time, international normalized ratio, hemoglobin concentration, creatinine, and several other critical measurements. A comprehensive list is provided in Multimedia Appendix 1.

### Statistical Methods

For descriptive analyses, continuous variables, such as laboratory indices, were summarized using medians and IQRs, while categorical variables were presented with frequencies and proportions. A 2-step variable selection process was used.

#### Univariate Analysis

Potential predictors of 14-day and 30-day readmissions were evaluated. Quantitative predictors were assessed using the Mann-Whitney *U* test, and binary predictors were analyzed with the chi-square test or the Fisher exact test. Considering the high dimensionality of the data and the complexity of the model, predictors with a *P* value less than .05 were retained for further analysis.

#### Automated Feature Selection

The selected predictors from the univariate analysis were then incorporated into an automatic variable selection process within the DES framework to identify the optimal subset of predictors. The final set of variables included in the model was determined based on a careful balance between accuracy and simplicity.

### The DES Framework

#### Key Features of the Framework

**Figure 1.**
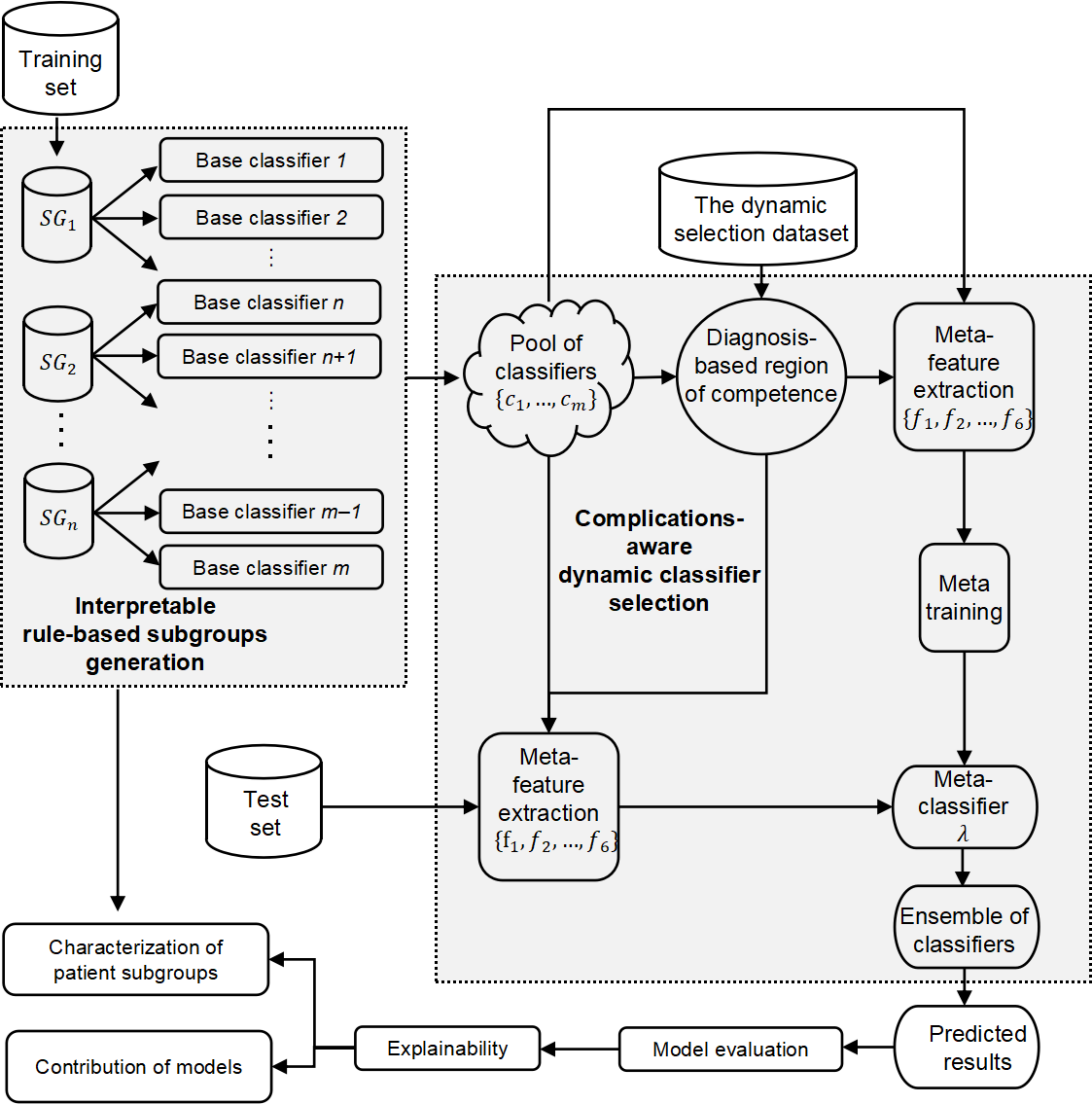
Overview of the proposed framework.

The proposed framework highlights 2 critical components: the generation of the classifier pool and the dynamic selection of classifiers. The classifiers trained with different training subsets play a pivotal role in tackling issues such as the incompleteness and sparsity of EHR data by capitalizing on the diverse feature selection capabilities across the heterogeneous classifiers. This approach enables the framework to maximize data utility even with fragmented or scarce data. Moreover, as patients’ health status evolves over time, the dynamic classifier selection process enables the framework to adapt more effectively to these fluctuations, enhancing its overall adaptability and relevance in real-world clinical scenarios. Notably, medical expert knowledge can be seamlessly integrated into both classifier pool generation and dynamic selection processes. This integration ensures that the framework is not only data-driven but also informed by the expertise and insights of medical professionals, adding a layer of clinical validity and practicality. An overview of the framework is graphically presented in Figure 1, providing a clear visual representation of its core components and interrelationships.

#### Classifier Pool Generation

Traditional DES systems typically generate diverse classifier pools through strategies such as altering initialization parameters, hyperparameter configurations, classifier models, training datasets, and feature subsets. In this study, to ensure the diversity of the base classifiers, we introduced a rule-based method to generate data subsets to support the training of classifiers. Given that complications are major causes of readmission in patients with cirrhosis as reported in existing studies, the descriptions of complications and comorbidities associated with liver cirrhosis are presented in Table 1. Based on this, we extracted rule sets that consist of binary variables representing medical diagnoses from real-world datasets. These rule sets allow us to characterize patient subgroups (SG_1_, SG_2_,..., SG_*n*_) based on distinct combinations of these complications and comorbidities. For example, a rule might be


(Electrolyte metabolic disorders=TRUE) AND (Hepatic encephalopathy=FALSE)

This rule-based method, grounded in medical diagnosis patterns, represents an innovative approach to integrating medical knowledge into the classifier generation process. By examining these subgroups, researchers and clinicians can gain deeper insights into the underlying reasons why individualized predictions are supported by classifiers trained on certain subsets of the data. By leveraging diverse data subsets and training heterogeneous classifiers, the classifier pool can capture a wide range of patterns within the EHR data to enhance the overall predictive performance. In this phase, a classifier pool $C=\left\{c_{1},c_{2},...,c_{m}\right\}$ was generated with *m* classifiers.

Besides diverse patient subgroups, a variety of machine learning algorithms can be integrated into the framework to create the classifier pool.

**Table 1.** Definition of complications and comorbidities.

Category	Complications	Comorbidities
Definition	Diseases or pathological conditions directly caused by liver cirrhosis	Other independent diseases or pathological conditions coexisting with the patient
Cause	Caused by liver cirrhosis (eg, portal hypertension and liver failure)	Caused by factors other than liver cirrhosis
Common stage	Most common in the decompensated stage	Can occur at any stage of the disease

#### Meta-Feature Extraction and Meta-Training

The DES problem involves selecting an optimal subset of base classifiers $C^{\prime }\subset C$ to classify a given test sample *x_j_*. To train a meta-classifier *λ* that acts as a classifier selector, the key challenge is to define the criteria to evaluate the competence of base classifiers as meta-features. In this study, 2 sets of meta-features are used. The first set of meta-features includes the criteria to measure the local accuracy in the region of competence (*f*_1_), extent of consensus in the region of competence (*f*_2_), overall accuracy in the region of competence (*f*_3_), accuracy in the decision space (*f*_4_), and degree of confidence for the input sample (*f*_5_). To extract those meta-features, a region of competence *θ_j_* is defined, for instance, *x_j_*, using the *k*-nearest neighbors clustering of the entire feature space of each data subset.

To effectively incorporate complications and comorbidities into the characterization of patients with cirrhosis and refine dynamic classifier selection, a novel competence region $\theta_{j}^{\prime }$ is defined. This region, grounded in medical diagnoses, emphasizes complications and comorbidities as binary indicators of their presence or absence. Notably, the competence region $\theta_{j}^{\prime }$ consists of an equal number of positive and negative data samples to ensure a balanced evaluation of the base classifiers’ performance. The new set of meta-features (*f*_6_) was defined as the local accuracy within the region of competence $\theta_{j}^{\prime }$ to be aware of patients’ subgroup with specific complications or comorbidities. Thus, a meta-feature vector is extracted as $v_{ij}=\left\{f_{1},f_{2},f_{3},f_{4},f_{5},f_{6}\right\}$ for classifier *c_i_* and instance *x_j_*. To construct the training set for the meta-classifier, labels are assigned to each meta-feature vector: for each meta-feature vector, its label $\alpha_{ij}=1$ if classifier *c_i_* correctly classifies instance *x_j_*, otherwise $\alpha_{ij}=0$. By embedding patients’ medical diagnoses, this framework proficiently captures the impact of complications and comorbidities during the dynamic selection of suitable classifiers. This approach represents an improvement over traditional methods, as it explicitly incorporates the unique characteristics of patients with cirrhosis. The critical role of this redefinition lies in its ability to determine model applicability for specific patient subgroups. Technical details of the implementation can be found in the GitHub repository [22]. This dynamic framework not only improves the predictive performance but also enhances the model’s adaptability to real-world clinical scenarios.

#### Dynamic Classifier Ensemble

In traditional machine learning workflows, a single model is typically used for both the training and testing datasets. Nevertheless, different models display diverse strengths and weaknesses in terms of accuracy, interpretability, and other aspects. The fundamental theory of dynamic classifier selection postulates that not every classifier in a pool is an expert for all unseen samples. On the contrary, each base classifier is proficient in distinct regions of the feature space. When we apply this concept to our study, it becomes clear that no single classifier can yield optimal prediction results for the entire test set. However, by using the dynamic classifier selection strategy, we are able to extract diverse meta-features for different data subsets and choose the base classifier that provides the most accurate prediction for each subset. This method guarantees the achievement of the optimal prediction results.

Given an instance $x_{j,test}$, the dynamic selection dataset, and the classifier pool, the set of meta-features $\left\{f_{1},f_{2},f_{3},f_{4},f_{5}\right\}$ is extracted using the region of competence *θ_j_*, and the meta-feature *f*_6_ is extracted using the region of competence $\theta_{j}^{\prime }$ based on the diagnosis of patients. Once the meta-features are extracted, the meta-classifier *λ* is used to calculate competence scores for each classifier in the pool. These competence scores, typically expressed as probabilities estimated by the meta-classifier, indicate the capability of each classifier. The classifiers with the highest competence score are selected as the pool of ensemble of classifiers C*. If multiple classifiers have equal competence scores, the predictions of those classifiers are aggregated using the majority vote rule.

## Results

### Data Analysis

The final dataset comprises 3307 patients with readmission records in this study. Among these patients, 1121 (33.9%) are female and 2186 (66.1%) are male, with an average age of 55 years. Within a 14-day period, 423 (12.8%) patients were readmitted, while within 30 days, 879 (26.6%) patients experienced readmission. Tables2  3 present detailed descriptive statistics for the entire dataset. Taking total bilirubin levels (normal range of 1.7‐21 µmol/L) as an example, it was observed that patients fulfilling the discharge criteria exhibited heightened total bilirubin levels. Upon admission, the percentages of patients with total bilirubin levels exceeding the normal range were 71.9% (304/423) for the 14-day period and 60.4% (531/879) for the 30-day period. However, at discharge, these percentages declined to 65.9% (279/423) and 54.7% (481/879), respectively. Both admission and discharge laboratory indicators aligned with the anticipated clinical outcomes for cirrhotic patients. The missing rates for all selected features are below 10%, and missing data were imputed using the mean of *k*-nearest neighbors.

**Table 2.** Characteristics of patients experiencing 14-day readmission.

Characteristics	Readmission (n=423)	Nonreadmission (n=2884)	*P* value
Demographics, n (%)			.006
Female	118 (27.9)	1003 (34.8)	
Male	305 (72.1)	1881 (65.2)	
Complications, n (%)			
Peritonitis	111 (26.2)	470 (16.3)	<.001
Hepatic encephalopathy	55 (13.0)	199 (6.9)	<.001
Abdominal fluid	55 (13.0)	232 (8.0)	.001
Liver failure	55 (13.0)	252 (8.7)	<.001
EMD^a^	49 (11.6)	230 (8.0)	.02
Ruptured esophagogastric varices	178 (42.1)	1603 (55.6)	<.001
Comorbidities, n (%)			
Autoimmune diseases	34 (8.0)	329 (11.4)	.07
Gastritis	28 (6.6)	316 (11.0)	.008
Neuropathy	22 (5.5)	75 (2.6)	.005
Ulcers	26 (6.2)	297 (10.3)	.009
Surgery, n (%)			
Bilirubin adsorption-plasma exchange	13 (3.1)	13 (0.5)	<.001
Bone marrow puncture biopsy	60 (14.2)	252 (8.7)	<.001
Autologous ascites concentration and reinfusion	48 (11.4)	88 (3.1)	<.001
Laboratory tests, median (IQR)			
Albumin	29.6 (26.50‐33.23)	31.7 (27.80‐36.20)	<.001
Erythrocyte count	3.13 (2.58‐3.62)	3.35 (2.80‐3.87)	<.001
Leukocyte count	4.59 (3.10‐6.89)	3.86 (2.74‐5.32)	<.001
Hemoglobin concentration	93 (74.00‐112.00)	100 (81.00‐119.00)	<.001
Total platelets	73 (46.00‐117.50)	63 (43.00‐100.00)	<.001
Total bilirubin	35.7 (17.55‐82.65)	24.75 (15.90‐41.50)	<.001
INR^b^	1.48 (1.28‐1.87)	1.34 (1.20‐1.55)	<.001
Prothrombin time	17.75 (15.70‐21.40)	16.4 (14.90‐18.60)	<.001
Creatinine	69.3 (57.10‐89.75)	65.7 (55.20‐0.40)	.002
Serum sodium	137.5 (132.90‐140.50)	139.3 (136.40‐141.80)	<.001
Serum potassium	4 (3.62‐4.40)	3.87 (3.57‐4.20)	<.001
Neutrophil count	3.14 (1.98‐5.23)	2.46 (1.64‐3.66)	<.001
Fibrinogen concentration	1.77 (1.27‐2.46)	2 (1.55‐2.62)	<.001
Urea nitrogen	6.02 (4.49‐9.38)	5.38 (4.17‐7.41)	<.001
Total protein	66.8 (56.75‐69.95)	66.2 (59.42‐72.57)	<.001
Prealbumin	65 (44.00‐95.50)	83 (54.00‐122.00)	<.001
Direct bilirubin	17.2 (8.70‐47.10)	11.5 (7.20‐21.60)	<.001
Indirect bilirubin	14.9 (8.30‐30.60)	11.8 (7.90‐18.70)	<.001
Total bile acids	39.1 (15.15‐94.30)	29.15 (12.30‐60.80)	<.001
Discharge laboratory tests, median (IQR)			
Albumin	30.1 (27.80‐33.00)	32.2 (29.40‐36.00)	<.001
Serum sodium	137.7 (134.00‐140.45)	139.2 (136.70‐141.45)	<.001
Total bilirubin	31.5 (16.25‐71.70)	23.5 (15.30‐38.30)	<.001
Total white blood cells	3.74 (2.42‐5.28)	3.4 (2.43‐4.63)	.006
INR	1.5 (1.29‐1.87)	1.34 (1.20‐1.57)	<.001
Creatinine	71.2 (56.35‐91.75)	68 (56.30‐83.80)	.02
Scoring variables, median (IQR)			
MELD^c^	13 (10.00‐18.00)	11 (8.00‐14.00)	<.001

a EMD: electrolyte metabolism disorder. It is an imbalance in the balance of electrolytes (eg, sodium, potassium, calcium, and chloride) in the body, usually caused by levels of electrolytes in the body that are outside of normal ranges.

b INR: international normalized ratio. INR = (PTpatient/PTcontrol)ISI, where PTpatient is the patient's prothrombin time, PTcontrol is the average normal prothrombin time, and ISI is the international sensitivity. The normal range of INR varies among hospitals, laboratories, and patient,s and ranges from 0.8 to 1.2.

c MELD: Model for End-Stage Liver Disease [23]. MELD = 9.57*ln(creatinine) + 3.78*ln(bilirubin) + 11.20*ln(INR) + 6.43. The patient's serum creatinine value is in milligrams per deciliter (mg/dL), the patient's total bilirubin value is in milligrams per deciliter (mg/dL), and the INR is the patient's international normalized ratio for coagulation. The higher the value, the higher the risk level.

**Table 3.** Characteristics of patients experiencing 30-day readmission.

Characteristics	Readmission (n=879)	Non-readmission (n=2428)	*P* value
Demographics, n (%)			.003
Female	262 (29.8)	859 (35.4)	
Male	617 (70.2)	1569 (64.6)	
Complications, n (%)			
Peritonitis	209 (23.8)	372 (15.3)	<.001
Hepatic encephalopathy	87 (9.9)	167 (6.9)	.005
Abdominal fluid	109 (12.4)	178 (7.3)	<.001
Liver failure	140 (15.9)	198 (8.2)	<.001
EMD^a^	90 (10.2)	189 (7.8)	.03
Ruptured esophagogastric varices	445 (50.6)	1336 (55.0)	.03
Comorbidities, n (%)			
Autoimmune diseases	70 (8.0)	293 (12.1)	<.001
Gastritis	67 (7.6)	277 (11.4)	.002
Neuropathy	35 (4.0)	62 (2.6)	.04
Ulcers	67 (7.6)	256 (10.5)	.02
Surgery, n (%)			
Bilirubin adsorption-plasma exchange	18 (2.1)	8 (0.3)	<.001
Bone marrow puncture biopsy	104 (11.8)	208 (8.6)	.006
Autologous ascites concentration and reinfusion	77 (8.8)	59 (2.4)	<.001
Laboratory tests, median (IQR)			
Albumin	30.1 (26.70‐33.70)	31.9 (27.90‐36.40)	<.001
Erythrocyte count	3.11 (2.60‐3.63)	3.38 (2.83‐3.89)	<.001
Leukocyte count	4.36 (3.02‐6.40)	3.8 (2.71‐5.28)	<.001
Hemoglobin concentration	93 (74.00‐113.00)	101 (82.00‐119.00)	<.001
Total platelets	71 (46.00‐115.00)	63 (43.00‐97.00)	<.001
Total bilirubin	29.1 (15.90‐64.25)	24.6 (16.10‐40.20)	<.001
INR^b^	1.42 (1.24‐1.72)	1.33 (1.20‐1.54)	<.001
Prothrombin time	17.2 (15.40‐20.20)	16.3 (14.90‐18.40)	<.001
Creatinine	68.9 (57.25‐87.10)	63.3 (55.00‐79.17)	<.001
Serum sodium	138 (134.00‐140.90)	139.4 (136.60‐141.90)	<.001
Serum potassium	3.92 (3.58‐4.34)	3.87 (3.57‐4.20)	<.001
Neutrophil count	2.93 (1.92‐4.71)	2.41 (1.59‐3.60)	<.001
Fibrinogen concentration	1.88 (1.40‐2.56)	2.02 (1.56‐2.61)	<.001
Urea nitrogen	5.96 (4.35‐9.04)	5.32 (4.14‐7.25)	<.001
Total protein	63.8 (58.00‐70.30)	66.3 (59.70‐72.88)	<.001
Prealbumin	70 (46.00‐109.00)	84 (55.00‐122.00)	<.001
Direct bilirubin	14.1 (7.70‐35.80)	11.4 (7.20‐21.10)	<.001
Indirect bilirubin	12.8 (7.70‐25.70)	11.8 (7.95‐18.30)	<.001
Total bile acids	32.3 (11.99‐79.16)	29.45 (12.80‐59.79)	.01
Discharge laboratory tests, median (IQR)			
Albumin	30.6 (28.40‐33.70)	32.4 (29.50‐36.10)	<.001
Serum sodium	138.1 (135.00‐140.78)	139.4 (137.00‐141.60)	<.001
Total bilirubin	27.4 (15.60‐54.80)	23.6 (15.40‐37.40)	<.001
Total white blood cells	3.66 (2.47‐5.06)	3.39 (2.41‐4.60)	.001
INR	1.42 (1.25‐1.75)	1.34 (1.20‐1.56)	<.001
Creatinine	70.85 (57.10‐90.10)	67.7 (56.00‐82.40)	<.001
Scoring variables, median (IQR)			
MELD^c^	12 (10.00‐17.00)	11 (8.00‐14.00)	<.001

a EMD: electrolyte metabolism disorder. It is an imbalance in the balance of electrolytes (eg, sodium, potassium, calcium, and chloride) in the body, usually caused by levels of electrolytes in the body that are outside of normal ranges.

b INR: international normalized ratio. INR = (PTpatient/PTcontrol)ISI, where PTpatient is the patient's prothrombin time, PTcontrol is the average normal prothrombin time, and ISI is the international sensitivity. The normal range of INR varies among hospitals, laboratories, and patients, and ranges from 0.8 to 1.2.

c MELD: Model for End-Stage Liver Disease [23]. MELD = 9.57*ln(creatinine) + 3.78*ln(bilirubin) + 11.20*ln(INR) + 6.43. The patient's serum creatinine value is in milligrams per deciliter (mg/dL), the patient's total bilirubin value is in milligrams per deciliter (mg/dL), and the INR is the patient's international normalized ratio for coagulation. The higher the value, the higher the risk level.

### Feature Selection and Statistical Analysis

A 2-step variable selection procedure was applied to the 14-day and 30-day readmission datasets, using tailored statistical methods for both quantitative and binary predictive factors. The application of these methods resulted in slightly different sets of included predictors for the 2 datasets. The *P* values for each variable are presented in Tables2  3. Predictive factors with *P* values less than .05 were subsequently considered in the automatic variable selection process within the DES framework, aimed at pinpointing the optimal subset of predictors. Selected variables for both datasets are detailed in MultimediaAppendices 2  3.

### The DES Framework Development

In the proposed DES framework, the classifier pool generation phase used an ensemble of 11 machine learning algorithms, each trained on different subsets and feature sets, to enhance the diversity and accuracy of trained models. During this phase, an interpretable set of rules was derived from each training dataset. Notably, these rules exhibited considerable overlap, suggesting the existence of common features across different rules. Across all training datasets, a total of 80 rules were extracted, with each rule covering at least 500 instances. These rules served as patterns for identifying specific characteristics or conditions within patient records. Using these rules, tailored data subsets were generated for experiments. Subsequently, during the meta-feature extraction and meta-training phase, 2 groups of meta-features were defined and extracted. Specifically, a customized competence region was designed to leverage the complications and comorbidities of cirrhotic patients, to extract meta-feature vectors for training the meta-classifier. Finally, in the dynamic classifier ensemble phase, the meta-classifier calculated the competence score of each classifier. Either the classifier with the highest score was selected, or a majority voting rule was applied to make predictions, ensuring optimal predictive performance in local regions for each patient.

The machine learning algorithms used to train the base classifiers include Naïve Bayes, *k*-nearest neighbors, logistic regression, linear discriminant analysis, quadratic discriminant analysis, and ensemble models such as random forest, extra trees, AdaBoost (adaptive boosting), gradient boosting classifier, LightGBM (light gradient boosting machine), and XGBoost (extreme gradient boosting). The hyperparameter tuning in this study comprised 2 components. First, the PyCaret library was used to automate machine learning workflows, including feature selection, model training, and hyperparameter optimization. Each model required tailored tuning metrics, such as the selection of the optimal *k*-value and distance metrics for *k*-nearest neighbors, tuning the *C* and *γ* parameters for the support vector machine, and adjusting the number and depth of trees for the random forest. Second, for the DES framework, hyperparameters such as the type of meta-classifier and the number of neighbors in local regions were optimized via grid search.

Furthermore, a 5-fold cross-validation approach was applied to maximize the use of a limited dataset, achieve stable predictions, and mitigate overfitting concerns. Building on the established framework of the DESlib library [24], the implementation of the proposed framework is accessible for online reference [22].

### Evaluation Results

**Figure 2.**
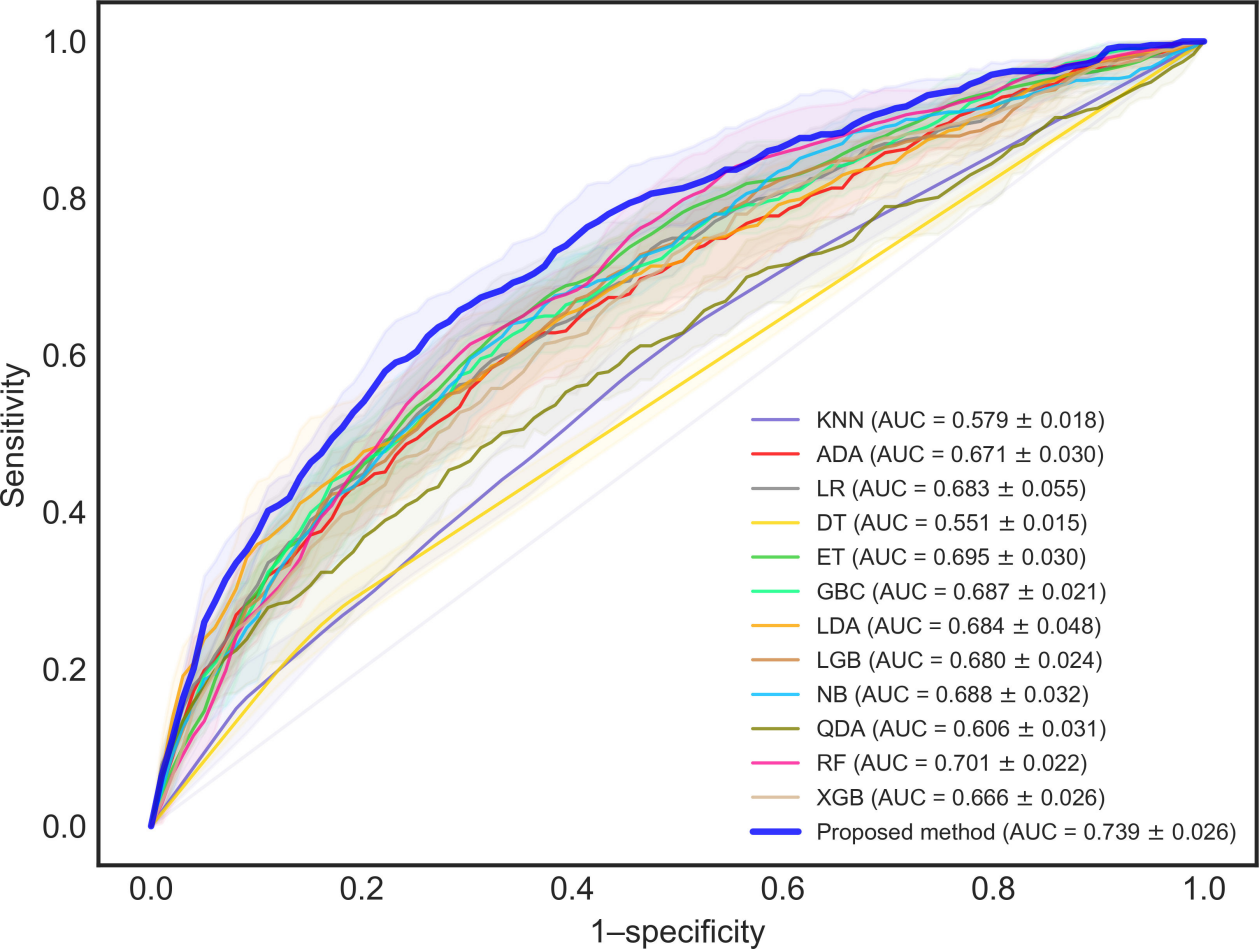
ROC (receiver operating characteristic) curves for the prediction of 14-day readmission. ADA: adaptive boosting; AUC: area under the receiver operating characteristic curve; DT: decision tree; ET: extra trees; GBC: gradient boosting classifier; KNN: *k*-nearest neighbors; LDA: linear discriminant analysis; LGB: light gradient boosting machine; LR: logistic regression; NB: Naive Bayes; QDA: quadratic discriminant analysis; RF: random forest; XGB: extreme gradient boosting.

The evaluation focused on assessing unplanned readmissions within 14 days and 30 days. To address the challenge of imbalanced classification, where the model might tend to predict the majority class, we implemented a sensitivity-focused threshold adjustment. The threshold used for calculating the accuracy score was chosen to ensure a sensitivity greater than 0.5, indicating a focus on correctly identifying positive cases. The AUROC metric quantifies the area under the receiver operating characteristic curve, which illustrates the relationship between the true positive rate and the false positive rate at various thresholds. Visual representations of these metrics are presented in Figures2  3. Table 4 summarizes the average scores obtained during cross-validation, alongside the maximum and minimum values observed. For 14-day readmission prediction, the DES framework achieved the highest accuracy of 0.783 and the peak AUC (area under the curve) of 0.739. For 30-day readmission prediction, it attained the best accuracy of 0.683 and the highest AUC of 0.684. In both accuracy and AUROC, the proposed method demonstrated superiority over the baseline models. This performance improvement can be attributed to the innovative features of our framework, such as the dynamic classifier selection based on patient subgroups.

**Figure 3.**
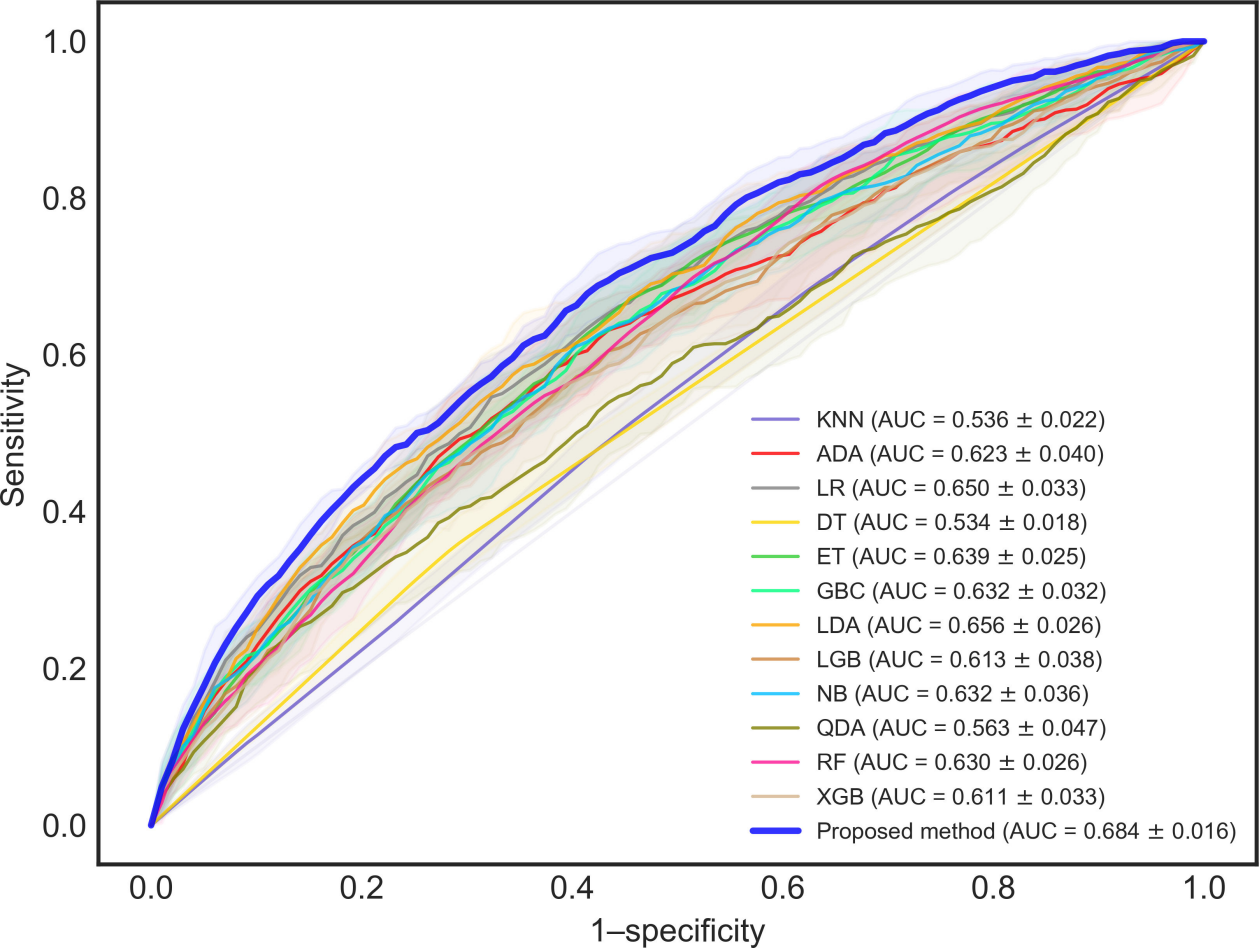
ROC (receiver operating characteristic) curves for the prediction of 30-day readmission. ADA: adaptive boosting; AUC: area under the receiver operating characteristic curve; DT: decision tree; ET: extra trees; GBC: gradient boosting classifier; KNN: *k*-nearest neighbors; LDA: linear discriminant analysis; LGB: light gradient boosting machine; LR: logistic regression; NB: Naive Bayes; QDA: quadratic discriminant analysis; RF: random forest; XGB: extreme gradient boosting.

**Table 4.** Performance comparison of different prediction models.

Classifier	14-day readmission		30-day readmission	
AUROC^a^	Accuracy	AUROC	Accuracy
AdaBoost^b^	0.671 (0.619‐0.706)	0.717 (0.635‐0.736)	0.623 (0.548‐0.659)	0.644 (0.590‐0.675)
Extra trees	0.695 (0.642‐0.727)	0.733 (0.682‐0.779)	0.639 (0.593‐0.662)	0.636 (0.581‐0.658)
Gradient boosting classifier	0.687 (0.648‐0.710)	0.729 (0.685‐0.791)	0.632 (0.579‐0.670)	0.635 (0.593‐0.657)
*K*-nearest neighbors	0.579 (0.558‐0.605)	0.557 (0.504‐0.589)	0.536 (0.502‐0.557)	0.507 (0.424‐0.569)
Linear discriminant analysis	0.684 (0.624‐0.761)	0.732 (0.657‐0.835)	0.656 (0.606‐0.681)	0.659 (0.578‐0.699)
LightGBM^c^	0.680 (0.644‐0.711)	0.718 (0.658‐0.772)	0.613 (0.546‐0.653)	0.623 (0.570‐0.679)
Logistic regression	0.683 (0.618‐0.763)	0.721 (0.604‐0.790)	0.650 (0.591‐0.681)	0.653 (0.584‐0.685)
Naïve Bayes	0.688 (0.641‐0.742)	0.735 (0.657‐0.802)	0.632 (0.560‐0.658)	0.642 (0.576‐0.676)
Quadratic discriminant analysis	0.606 (0.548‐0.631)	0.647 (0.623‐0.672)	0.563 (0.492‐0.628)	0.580 (0.501‐0.672)
Random forest	0.701 (0.668‐0.726)	0.728 (0.711‐0.748)	0.630 (0.586‐0.655)	0.620 (0.606‐0.634)
XGBoost^d^	0.666 (0.624‐0.702)	0.694 (0.622‐0.739)	0.611 (0.561‐0.660)	0.616 (0.563‐0.678)
Proposed method	0.739 (0.697‐0.775)	0.783 (0.766‐0.803)	0.684 (0.664‐0.709)	0.683 (0.658‐0.701)

a AUROC: area under the receiver operating characteristic curve.

b AdaBoost: adaptive boosting.

c LightGBM: light gradient boosting machine.

d XGBoost: extreme gradient boosting.

**Figure 4.**
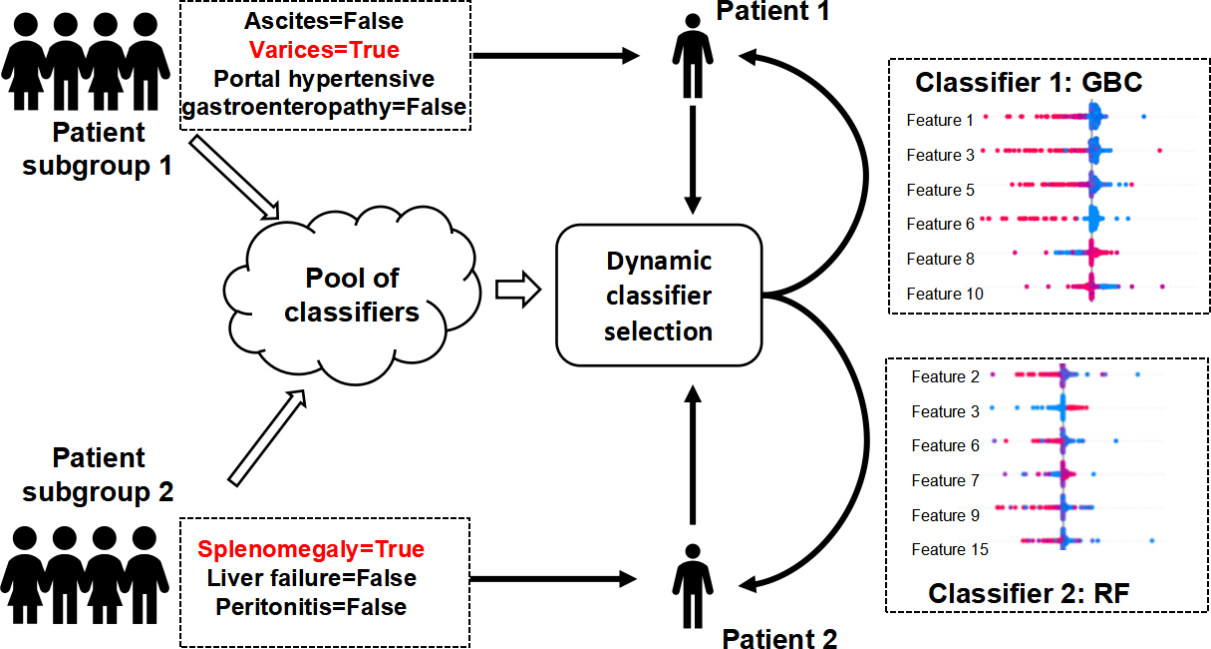
Illustration of characterization of patient subgroups and dynamic model selection for different patients. GBC: gradient boosting classifier; RF: random forest.

Although the accuracy of the model is not particularly high, a result similar to those reported in comparable literature, it was found that in some patient subgroups, the framework we used can exhibit more promising application prospects. This also constitutes one of the significant values of our research. Data-driven models are challenging to implement in real-world clinical applications due to their potential lack of generalizability across all patient populations. Within our framework, during the process of dynamic classifier selection, we can initially ascertain whether a model is suitable for a specific patient. This is accomplished by evaluating the performance of models with respect to the corresponding patient subgroups. As illustrated in Figure 4, patient subgroup 1 consists of patients with liver cirrhosis who have varices but no ascites or portal hypertensive gastroenteropathy. Patient subgroup 2 comprises patients with liver cirrhosis who have splenomegaly but no liver failure or peritonitis. Multiple heterogeneous classifiers were trained using multiple data subsets that meet these criteria to form the classifier pool. For patients 1 and 2, a gradient boosting classifier and a random forest classifier were selected, respectively. We evaluated the performance of the aforementioned subgroups. For the patients (n=152) in the test set who fit the criteria of subgroup 1, the accuracy, precision, recall, and *F*_1_-score were 0.881, 0.862, 0.881, and 0.870, respectively. For the patients (n=195) in the test set who met the criteria of subgroup 2, the accuracy, precision, recall, and *F*_1_-score were 0.891, 0.872, 0.891, and 0.879, respectively.

### Interpretability

In addition to improving accuracy, the proposed method offers the capability to generate a classifier pool and facilitate dynamic selection of classifiers in a highly interpretable manner [25]. The interpretable rules used to generate the training subset for this classifier can describe clinical characteristics of patient subgroups. These rules provide insights into the specific features or conditions that contribute to the prediction, thereby assisting clinicians in making informed decisions and tailoring interventions for different patient subgroups. In cases where multiple classifiers are selected for prediction, analyzing the intersection of elements across these rules can provide additional insights for characterizing patient subgroups.

**Figure 5.**
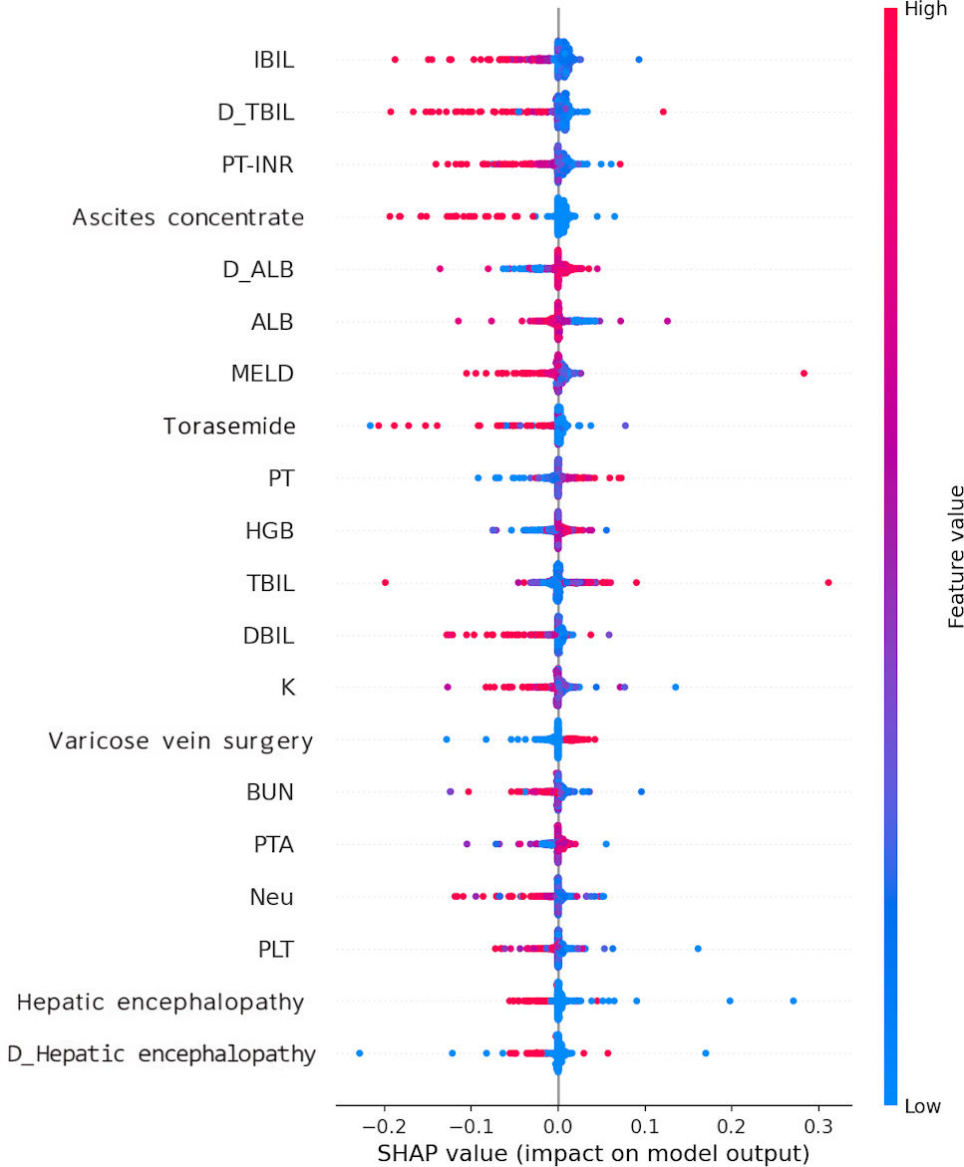
SHAP summary plot of the effect of input variables for 14-day readmission. ALB: albumin; BUN: blood urea nitrogen; D_ALB: albumin at discharge; D_Hepatic encephalopathy: hepatic encephalopathy at discharge; D_TBIL: total bilirubin at discharge; DBIL: bilirubin at discharge; HGB: hemoglobin; IBIL: indirect bilirubin; K: potassium; MELD: Model for End-Stage Liver Disease; Neu: neutrophils; PLT: platelet count; PT: prothrombin time; PTA: prothrombin activity; PT-INR: prothrombin time – international normalized ratio; SHAP: Shapley Additive Explanations; TBIL: total bilirubin.

**Figure 6.**
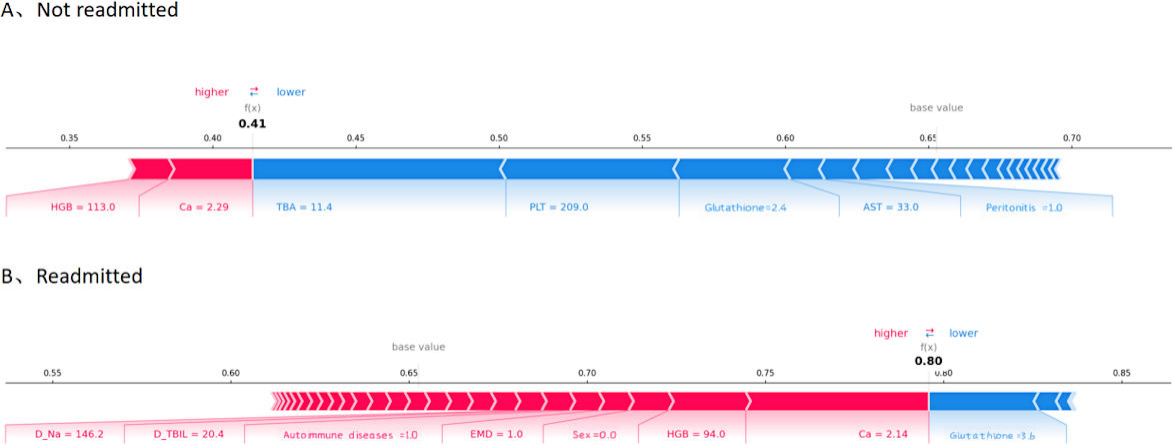
SHAP (Shapley Additive Explanations) force plot showing (A) not readmitted and (B) readmitted. AST: aspartate aminotransferase; Ca: calcium; D_Na: sodium at discharge; D_TBIL: total bilirubin at discharge; EMD: electrolyte metabolism disorder; HGB: hemoglobin; PLT: platelet count; TBA: total bile acid.

During the dynamic classifier selection phase, the proposed method selects classifiers with the highest competence scores for prediction. Our experiments revealed that, in most instances, a single optimal classifier was chosen for each individual to predict the risk of readmission. When the selected classifier is inherently intelligible and explainable, its predictions can be directly interpreted and understood. If multiple classifiers or black box models are selected for prediction, interpretability techniques such as SHAP (Shapley Additive Explanations) can be used to interpret the model’s predictions. For instance, Figure 5 demonstrates that higher indirect bilirubin, total bilirubin at discharge, prothrombin time – international normalized ratio, ascites concentrate, and lower albumin at discharge were associated with higher predicted outcomes for a patient subgroup. Figure 6 presents individual SHAP force diagrams for non-readmitted and readmitted patients, with predicted probabilities of 0.41 and 0.80 for patients 1 and 2, respectively. For the first patient, total bile acid, platelet count, glutathione, aspartate aminotransferase, and peritonitis contributed negatively to the predicted outcome, while calcium and hemoglobin contributed positively. Conversely, for the second patient, calcium, hemoglobin, sex, electrolyte metabolism disorder, autoimmune diseases, total bilirubin at discharge, and sodium at discharge contributed positively to the prediction, while glutathione contributed negatively.

## Discussion

In this study, we developed a DES framework that comprises 3 components: classifier pool generation, meta-feature extraction and meta-training, and dynamic classifier ensemble. Initially, the classifier pool was formed by generating data subsets using a rule-based method to characterize patient subgroups and train heterogeneous machine learning models. Subsequently, by defining competence regions that encompass both complications and comorbidities, meta-features were extracted, and a meta-classifier was trained. Finally, a dynamic classifier ensemble was used to select the optimal classifier based on competent scores calculated by the meta-classifier, while a majority voting strategy was used when deemed necessary. This framework demonstrates its potential for application in EHR-based predictive tasks, by adaptively selecting models for individuals, enhanced by the medical knowledge integrated into the DES framework. The dynamic alignment of classifier selection with diverse patient subgroups, identified as the core contribution of this research, significantly enhances the clinical applicability and reliability of the model. By capturing heterogeneous health profiles and treatment responses across patient populations, the framework accommodates the complexity of real-world clinical care, thereby facilitating clinical experts’ comprehension and trust in model predictions.

Readmission, a critical indicator of hospital quality [26], performance [27], and patient care standards [12], imposes significant burdens on health care systems and costs, with nationwide readmission expenses surpassing US $4.45 billion annually [28] and posing substantial survival challenges for low-income families [29]. Accurate prediction of readmission is increasingly essential for the effectiveness of interventions aimed at reducing readmissions. While bed availability may influence the timing of readmissions, it is not a determinant of readmission occurrence itself. In China’s highly efficient health care system, most patients requiring treatment can access timely care, and bed availability variations (eg, overcrowding vs idleness in specialized units) were not major factors in the hospitals included in this study. To address potential timing biases, this research reports predictions for 2 discrete time intervals (14-day and 30-day readmission), providing comprehensive coverage of readmission dynamics [30]. Although bed availability could theoretically be a confounding factor, its impact was minimized in our analysis.

Over the past decade, numerous studies have modeled readmission risks using diverse methods, typically reporting AUC scores within the range of 0.60 to 0.78 [10,14,15]. The majority of studies have focused on developing and validating a single machine learning model. In contrast, within our study, the proposed DES method demonstrated superiority over other approaches. Notably, the framework implicitly addressed class imbalance through subgroup-specific undersampling during classifier pool generation, whereas explicit techniques such as SMOTE (Synthetic Minority Oversampling Technique) did not improve performance in our experiments.

Our study revealed that the 14-day unplanned readmission rate for patients with cirrhosis was 12.8% (423/3307), while the 30-day readmission rate stood at 26.6% (879/3307). In contrast, the 30-day readmission rates among insurance-covered populations in 2014 and 2019 were 22.6% and 21%, respectively [7]. The slightly elevated readmission rates observed in our study may be attributed to the higher average patient age of 55 years, which is associated with increased disease severity and a greater propensity for comorbidities. These patients are also more susceptible to developing complications and infections during hospitalization [2]. According to weighted analyses of readmission databases, complications [31], MELD (Model for End-Stage Liver Disease) scores [7], and infections [28] are crucial predictors of readmission in patients with cirrhosis. Our study echoed these findings, with prevalent complications including ascites (14-day data: 368 cases vs 55 controls; 30-day data: 770 cases vs 106 controls), variceal bleeding (14-day: 245 vs 178; 30-day: 434 vs 445), hepatic encephalopathy (reference 21; 14-day: 368 vs 55; 30-day: 792 vs 87), spontaneous bacterial peritonitis (14-day: 312 vs 111; 30-day: 670 vs 209), and acute-on-chronic liver failure (14-day: 337 vs 86; 30-day: 739 vs 140). These results are largely in alignment with previous studies [32]. Our study further revealed a gender disparity in liver disease prevalence, with male patients comprising the majority of readmissions (14-day: 305 vs 118; 30-day: 617 vs 262). This may be linked to unhealthy lifestyle behaviors, including a higher prevalence of alcohol-related cirrhosis in males [33] and increased susceptibility to infections, both of which contribute to elevated readmission rates. Smoking, another established risk factor for cirrhosis, underscores the need for early intervention targeting smoking and alcohol cessation in cirrhotic patients. However, the asymptomatic nature of early-stage cirrhosis often delays diagnosis, exacerbating disease severity and readmission risks in males.

The advantages of this study can be summarized as follows. First, by leveraging heterogeneous classifiers’ diverse feature selection capabilities and integrating temporal dynamics of patient health status into the DES framework, the model’s robustness and clinical applicability are enhanced to address real-world EHR data limitations. Second, medical expert knowledge is seamlessly integrated into both classifier pool generation and dynamic selection processes. Specifically, medical diagnosis-based rules are used to define patient subgroups and competence regions, ensuring the framework is not purely data-driven but also informed by clinical expertise. This integration adds clinical validity—for example, incorporating complications and comorbidities (critical factors in clinical assessments)—to enhance model relevance and interpretability in real-world practice. Third, the framework enables dynamic updates of classifier pools and meta-classifiers with incoming data, demonstrating flexibility and scalability that hold promise for extending its application to prediction and management of a broad spectrum of diseases.

This study acknowledges several limitations. First, the dataset used in this study does not include patients readmitted from other hospitals. Although previous research suggests that the majority of readmissions occur within the same institution, this limitation may potentially affect the comprehensiveness of the study. Additionally, the study only encompasses patients who have had at least 2 readmissions. Future research should strive to expand the dataset and the scope of the sample to improve the reliability and generalizability of the findings. Second, the risk of readmission is affected by a multitude of factors, including socioeconomic status, educational level, and health insurance. This complexity poses a certain constraint on the model’s discriminatory power. In future research, the goal is to incorporate these indicators through prospective and follow-up studies, thereby enhancing the model’s predictive accuracy. Third, the practical implementation of the model requires continuous monitoring and calibration in line with local medical environments. Future studies should give priority to developing methods to enhance data interoperability across different sites, which will facilitate a wider application of the model.

## Supplementary material

10.2196/63581 Multimedia Appendix 1 All variables in the dataset collected in the study.

10.2196/63581 Multimedia Appendix 2 All variables in the 14-day readmission prediction dataset.

10.2196/63581 Multimedia Appendix 3 All variables in the 30-day readmission prediction dataset.

## Abbreviations

AdaBoost: adaptive boosting
AUC: area under the curve
AUROC: area under the receiver operating characteristic curve
DES: dynamic ensemble selection
EHR: electronic health record
ICD-10: *International Classification of Diseases*, 10th Revision
ICD-9: *International Classification of Diseases*, 9th Revision
LightGBM: light gradient boosting machine
MELD: Model for End-Stage Liver Disease
META-DES: dynamic ensemble selection using meta-learning
SHAP: Shapley Additive Explanations
SMOTE: Synthetic Minority Oversampling Technique
TRIPOD: Transparent Reporting of a Multivariable Prediction Model for Individual Prognosis or Diagnosis
XGBoost: extreme gradient boosting
